# Pembrolizumab-Induced Addison’s Disease Leading to Severe Hyponatremia in a Breast Cancer Survivor: A Case Report With Implications for Emergency Department Practice

**DOI:** 10.7759/cureus.71759

**Published:** 2024-10-18

**Authors:** Ömerul F Aydin

**Affiliations:** 1 Medicine, Istanbul Yeni Yuzyil University, Istanbul, TUR

**Keywords:** addison, breast cancer, emergency, hyponatremia, pembrolizumab

## Abstract

Pembrolizumab, a PD-1 inhibitor, has become a cornerstone in the treatment of various cancers, including breast cancer. However, its use is associated with immune-related adverse effects (irAEs), particularly those involving the endocrine system. This case report presents a rare instance of pembrolizumab-induced Addison’s disease leading to severe hyponatremia. The case is supported by detailed laboratory findings and evaluated using the Naranjo adverse drug reaction probability scale. A 53-year-old female with a history of breast cancer presented with dizziness and fatigue while on a cruise. Initial laboratory tests revealed severe hyponatremia (serum sodium 117 mEq/L). Further evaluation revealed low cortisol (1.7 µg/dL) and elevated adrenocorticotropic hormone (ACTH) (452 pg/mL), indicative of adrenal insufficiency. Although thyroid function was normal, low IGF-1 levels suggested secondary adrenal insufficiency. The administration of hydrocortisone resulted in rapid symptom improvement, and the patient was discharged with a prescription for ongoing corticosteroid therapy. The Naranjo scale score of 4 indicated a possible relationship between pembrolizumab and the development of Addison’s disease. This case underscores the critical need for awareness of irAEs in patients undergoing treatment with immune checkpoint inhibitors. The application of the Naranjo scale provided a quantitative assessment of the likelihood that pembrolizumab induced adrenal insufficiency. Emergency department protocols should incorporate endocrine evaluations for patients presenting with non-specific symptoms while undergoing immunotherapy. Pembrolizumab can lead to severe endocrine disorders, such as Addison's disease, which can result in life-threatening conditions like severe hyponatremia. Emergency clinicians must remain vigilant in recognizing and treating these adverse effects to optimize patient outcomes.

## Introduction

Pembrolizumab, an immune checkpoint inhibitor that targets the programmed death-1 (PD-1) receptor, is a pivotal treatment option for a range of malignancies, including melanoma, non-small cell lung cancer, and, more recently, breast cancer. By blocking PD-1, pembrolizumab augments T-cell activity against tumor cells, but this enhancement of the immune response can inadvertently trigger immune-related adverse effects (irAEs), particularly within the endocrine system [1,2].

Among these irAEs, Addison’s disease, a form of primary adrenal insufficiency, is particularly concerning. This condition arises from autoimmune destruction of the adrenal cortex, leading to deficiencies in cortisol and aldosterone. Such deficiencies can precipitate life-threatening electrolyte disturbances, notably hyponatremia [3]. This report presents a case of a breast cancer patient who developed Addison’s disease, resulting in severe hyponatremia while receiving pembrolizumab. We also explore the application of the Naranjo adverse drug reaction probability scale to this case, providing a structured approach to assessing the likelihood of drug-induced adverse events [4].

## Case presentation

Patient history

The patient is a 53-year-old female with a history of triple-negative breast cancer (TNBC). The tumor biology was confirmed as estrogen receptor (ER) negative, progesterone receptor (PR) negative, and HER2 negative, which aligns with the TNBC subtype, as diagnosed at the oncology center where she was being followed. She had undergone 16 cycles of chemotherapy, including agents such as doxorubicin and cyclophosphamide, followed by taxane-based therapy. After achieving remission, pembrolizumab was initiated as part of her maintenance therapy. By the time of the syndrome of inappropriate antidiuretic hormone secretion (SIADH) onset, the patient had completed eight cycles of pembrolizumab. Notably, the patient had no prior history of adrenal insufficiency or other significant endocrine disorders [1].

Clinical presentation

While on a cruise, the patient began experiencing symptoms of dizziness, fatigue, and shortness of breath. Initial evaluation aboard the cruise ship revealed severe hyponatremia (serum sodium 117 mEq/L). The patient was stabilized with intravenous fluids and subsequently transferred to a hospital for further evaluation [2].

Upon arrival at the emergency department, her vital signs included a blood pressure of 90/60 mmHg and a heart rate of 75 beats per minute. Physical examination revealed that the patient was alert, oriented, and without signs of dehydration. The Glasgow Coma Scale score was 15, indicating full consciousness. In the evaluation of hyponatremia etiology, adrenocorticotropic hormone (ACTH) levels were found to be significantly elevated, while cortisol levels were observed to be low (Table 1).

**Table 1:** Laboratory test results of the patient and normal reference ranges of the values mEq/L: milliequivalents per liter; µg/dL: micrograms per deciliter; pg/mL: picograms per milliliter; ng/mL: nanograms per milliliter; µIU/mL: micro-international units per milliliter; pmol/L: picomoles per liter; mOsm/kg: milliosmoles per kilogram; H2O: water; ACTH: adrenocorticotropic hormone; TSH: thyroid stimulating hormone

Laboratory Test	Result	Reference Range
Serum sodium	117 mEq/L	135-145 mEq/L
Serum cortisol	1.7 µg/dL	3.7-19.4 µg/dL
ACTH	452 pg/mL	10-60 pg/mL
IGF-1 (somatomedin C)	137 ng/mL	Varies by age
Serum potassium	4.9 mEq/L	3.5-5.0 mEq/L
Serum creatinine	0.74 mg/dL	0.6-1.3 mg/dL
Serum urea	49 mg/dL	7-20 mg/dL
TSH	2.17 µIU/mL	0.4-4.0 µIU/mL
T3	4.18 pmol/L	3.1-6.8 pmol/L
T4	1.32 pmol/L	0.7-1.8 pmol/L
Serum osmolality	260 mOsm/kg	275-295 mOsm/kg
Urine osmolality	799 mOsm/kg H2O	300-900 mOsm/kg H2O

Diagnosis and treatment

The combination of low serum cortisol, elevated ACTH, and severe hyponatremia strongly suggested primary adrenal insufficiency, specifically Addison’s disease [3]. The patient’s normal thyroid function and relatively low IGF-1 levels pointed to possible pituitary involvement, which could be secondary to an autoimmune process triggered by pembrolizumab [4].

As part of the patient’s routine follow-up at the oncology center, a comprehensive PET-CT scan was performed prior to this episode. The PET-CT did not reveal any evidence of new primary or secondary tumors, including adrenal involvement. Given the high sensitivity of PET-CT in detecting metabolic activity, it was deemed sufficient to rule out the presence of malignancy, and therefore, a specific CT scan to exclude a primary tumor was not considered necessary. This clarification will be added to the manuscript.

The patient was treated with intravenous hydrocortisone, which served as an effective antagonist to the adrenal insufficiency (acting as an antidote). Sodium replacement therapy was also initiated. The patient’s symptoms improved rapidly, and her serum sodium levels stabilized. She was discharged with instructions to continue oral hydrocortisone and was advised to follow up with an endocrinologist for further management [5].

Naranjo adverse drug reaction probability scale

To assess the likelihood that pembrolizumab was responsible for the development of Addison’s disease and the subsequent hyponatremia, the Naranjo adverse drug reaction probability scale [6] was applied (Table 2).

**Table 2:** Naranjo adverse drug reaction probability scale for this case ACTH: adrenocorticotropic hormone Naranjo scale: A method for estimating the probability of adverse drug reactions, as described by Naranjo et al. (1981) in Clinical Pharmacology & Therapeutics [5]

Question	Answer	Score
1. There are previous conclusive reports on this reaction.	Yes	+1
2. Did adverse events occur after the suspected drug was administered?	Yes	+2
3. Did the adverse reactions improve when the drug was discontinued or a specific antagonist (hydrocortisone) was administered?	Yes	+1
4. Did the adverse event reappear when the drug was re-administered?	Unknown	0
5. Are there alternative causes (other than the drug) that could cause the reaction on their own?	Yes	-1
6. Did the reaction reappear when the placebo was administered?	Unknown	0
7. Was the drug detected in the blood (or other fluids) in concentrations known to be toxic?	No	0
8. The reaction was more severe when the dose was increased and less severe when the dose was decreased.	Unknown	0
9. Did the patient have a similar reaction to the same or similar drugs during any previous exposure?	No	0
10. Was the adverse event confirmed by objective evidence?	Yes	+1
Total Score		4

Interpretation: The total score of 4 suggests a "possible" adverse reaction to pembrolizumab, indicating a reasonable basis to suspect the drug as the cause of Addison’s disease, although other factors may also have played a role. The rapid improvement following hydrocortisone therapy strengthens the association between pembrolizumab and the adverse event [3,6].

## Discussion

The case presented highlights a rare but critical adverse effect of pembrolizumab: the development of Addison’s disease leading to severe hyponatremia. Pembrolizumab, by inhibiting the PD-1 pathway, effectively enhances the immune system’s ability to target and destroy tumor cells. However, this same mechanism can inadvertently trigger autoimmune reactions against normal tissues, including the adrenal glands. The result is a cascade of immune-mediated destruction, particularly of the adrenal cortex, leading to cortisol and aldosterone deficiencies, hallmark features of Addison’s disease [1].

In the context of this case, the elevated ACTH levels despite low cortisol concentrations strongly suggest that the patient’s adrenal glands were failing to produce adequate cortisol in response to the body’s demand, a condition exacerbated by pembrolizumab-induced autoimmunity. This pathophysiological mechanism aligns with previous reports of immune checkpoint inhibitors inducing adrenal insufficiency through similar pathways [1].

The development of adrenal insufficiency as an immune-related adverse effect (irAE) of immune checkpoint inhibitors is well-documented, though it remains a relatively rare occurrence. Vandiver et al. (2016) described a case of severe hyponatremia associated with immune nephritis following nivolumab administration, another PD-1 inhibitor. Although the primary adverse effect was nephritis, the occurrence of hyponatremia parallels the electrolyte disturbance observed in our patient, underscoring the broad spectrum of potential irAEs associated with PD-1 inhibitors [3].

Similarly, Hodi et al. (2010) reported the efficacy and adverse effects of ipilimumab, a CTLA-4 inhibitor, in metastatic melanoma patients. While ipilimumab targets a different checkpoint pathway than pembrolizumab, the study highlighted the potential for severe endocrine dysfunctions, including hypophysitis, which shares a pathophysiological overlap with adrenal insufficiency. The insights from this study support the broader understanding that immune checkpoint inhibitors, regardless of the specific target, can induce significant endocrine disturbances [4].

The Naranjo adverse drug reaction probability scale, utilized in our case to assess the likelihood of pembrolizumab being the causative agent, has been a valuable tool in previous studies. The scale provided a structured framework for evaluating drug-induced adverse events, as initially proposed by Naranjo et al. (1981). In our patient, a score of 4 indicated a “possible” relationship between pembrolizumab and the onset of Addison’s disease, consistent with the method’s application in other contexts where causality is uncertain but plausible [5].

Furthermore, Robert et al. (2015) explored the use of nivolumab in untreated melanoma patients, reporting various irAEs, including endocrine dysfunctions. The overlap in the types of irAEs observed with nivolumab and pembrolizumab suggests a class effect among PD-1 inhibitors, which may predispose patients to similar autoimmune sequelae. This reinforces the need for vigilance in monitoring patients for such adverse effects, particularly in those undergoing long-term immunotherapy [6].

From a clinical perspective, this case underscores the importance of early recognition and intervention in patients presenting with non-specific symptoms such as fatigue, dizziness, or electrolyte imbalances, especially in the setting of immunotherapy. The rapid identification of Addison’s disease and the prompt initiation of hydrocortisone therapy were crucial in stabilizing the patient’s condition and preventing further complications [3].

Emergency departments (EDs) play a pivotal role in the initial management of irAEs, particularly when they present acutely, as in this case. A thorough medication history is essential to identifying potential links between the patient’s symptoms and their ongoing treatment with immune checkpoint inhibitors. Moreover, the application of standardized tools like the Naranjo scale can assist clinicians in assessing the likelihood of a drug-induced event, guiding appropriate therapeutic interventions [5].

## Conclusions

In conclusion, this case report adds to the growing body of evidence linking pembrolizumab and other immune checkpoint inhibitors with severe endocrine-related irAEs, such as Addison’s disease. The case highlights the need for ongoing research into the mechanisms underlying these adverse effects, as well as the development of strategies to predict and mitigate them. Future studies should focus on identifying patient-specific risk factors that may predispose individuals to such adverse events, thereby enhancing the safety and efficacy of immune checkpoint inhibitors in oncology practice.

Emergency physicians and oncologists alike must maintain a high index of suspicion for irAEs in patients undergoing immunotherapy, ensuring that such adverse effects are promptly recognized and effectively managed to improve patient outcomes.

## References

[1] Kurokawa K, Mitsuishi Y, Shimada N (2023). Clinical characteristics of adrenal insufficiency induced by pembrolizumab in non-small-cell lung cancer. Thorac Cancer.

[2] Fujimiya T, Azuma K, Togashi Y, Kuwata K, Unezaki S, Takeuchi H (2024). Pembrolizumab-induced secondary adrenal insufficiency due to adrenocorticotrophic hormone deficiency in a patient with non-small-cell lung carcinoma: a case report. J Pharm Health Care Sci.

[3] Vandiver JW, Singer Z, Harshberger C (2016). Severe hyponatremia and immune nephritis following an initial infusion of nivolumab. Target Oncol.

[4] Hodi FS, O'Day SJ, McDermott DF (2010). Improved survival with ipilimumab in patients with metastatic melanoma. N Engl J Med.

[5] Romach MK, Piafsky KM, Abel JG, Khouw V, Sellers EM (1981). Methadone binding to orosomucoid (alpha 1-acid glycoprotein): determinant of free fraction in plasma. Clin Pharmacol Ther.

[6] Robert C, Long GV, Brady B (2015). Nivolumab in previously untreated melanoma without BRAF mutation. N Engl J Med.

